# Vitamin D supplementation and selected metabolic parameters in patients with type 2 diabetes and obesity: a prospective observational study

**DOI:** 10.3389/fendo.2025.1750040

**Published:** 2026-01-14

**Authors:** Karolina Hoffmann, Wiesław Bryl, Bhoomendra Bhongade, Ashot Avagimyan, Mohammed El-Tanani, Syed Arman Rabbani, Sirajunisa Talath, Imran Rashid Rangraze, Adil Farooq Wali, Walaa Ibraheem, Shakta Mani Satyam, Sorina Ispas, Ioannis Ilias, Viviana Maggio, Manfredi Rizzo, Anna Paczkowska

**Affiliations:** 1 Department and Clinic of Internal Diseases and Metabolic Disorders, Poznan University of Medical Sciences, Poznań, Poland; 2 Department of Pharmaceutical Chemistry, Ras Al Khaimah Medical and Health Sciences University, Ras Al Khaimah, United Arab Emirates; 3 Department of Internal Diseases Propedeutics, Yerevan State Medical University after M. Heratsi, Yerevan, Armenia; 4 Department of Clinical Pharmacy and Pharmacology, RAK College of Pharmacy, RAK Medical and Health Sciences University, Ras Al Khaimah, United Arab Emirates; 5 Department of Pharmacology, RAK College of Medical Sciences, RAK Medical and Health Sciences University, Ras Al Khaimah, United Arab Emirates; 6 Department of Internal Medicine, RAK College of Medicine, RAK Medical and Health Sciences University, Ras Al Khaimah, United Arab Emirates; 7 RAK College of Pharmacy, RAK Medical and Health Sciences University, Ras Al Khaimah, United Arab Emirates; 8 Department of Anatomy, Faculty of Medicine, Ovidius University, Constanta, Romania; 9 Department of Endocrinology, Hippokration Hospital, Athens, Greece; 10 School of Medicine, Promise Department, University of Palermo, Palermo, Italy; 11 Ras Al Khaimah Medical and Health Sciences University, Ras Al Khaimah, United Arab Emirates; 12 Department of Pharmacoeconomics and Social Pharmacy, Poznan University of Medical Sciences, Poznań, Poland

**Keywords:** glycemic control, metabolic parameters, obesity, type 2 diabetes, vitamin D deficiency, vitamin D supplementation

## Abstract

**Introduction:**

Vitamin D deficiency has been implicated in metabolic dysregulation, including insulin resistance and inflammation, commonly observed in patients with type 2 diabetes mellitus (T2DM) and obesity. Evidence on the metabolic impact of vitamin D supplementation in this population remains inconsistent.

**Objective:**

To evaluate the effects of high-dose vitamin D3 supplementation on anthropometric and selected metabolic parameters in ambulatory obese patients with T2DM treated with metformin monotherapy.

**Methods:**

This 12-week prospective cohort study included 200 patients with T2DM, allocated to a supplementation group (n = 100; vitamin D3 - 4,000 IU/day) or a control group (n = 100; no supplementation). Primary outcome was change in serum 25-hydroxyvitamin D [25(OH)D] concentration. Secondary outcomes included fasting serum glucose (FSG), glycated hemoglobin (HbA1c), blood pressure (BP), serum calcium, and body mass index (BMI). Predictors of failure to achieve target HbA1c ≤ 6.5% were identified using logistic regression.

**Results:**

After 12 weeks, serum 25(OH)D significantly increased in the supplementation group compared with controls (Δ +23.7 vs +1.3 ng/mL; p < 0.001). FSG and HbA1c decreased significantly in the intervention group (Δ –0.4 mmol/L, p = 0.02; Δ –0.6%, p = 0.01, respectively), while no significant changes were observed in systolic or diastolic BP, serum calcium, or BMI. Logistic regression identified higher baseline FSG (OR 1.34, 95% CI 1.12–1.61), longer diabetes duration (OR 1.28, 95% CI 1.07–1.54), and higher BMI (OR 1.21, 95% CI 1.01–1.47) as independent predictors of suboptimal glycemic response.

**Conclusions:**

High-dose vitamin D3 supplementation significantly improved vitamin D status and was associated with modest improvements in glycemic control in obese patients with T2DM, without affecting blood pressure, calcium, or body weight. These findings support vitamin D repletion as a potential adjunctive strategy in diabetes management, while not allowing causal inference, and warrant further confirmation in randomized controlled trials with longer follow-up.

## 1 Introduction

Since the 1990s, observational studies have consistently shown that serum 25-hydroxyvitamin D [25(OH)D] concentrations are inversely associated with type 2 diabetes mellitus (T2DM) and features of the metabolic syndrome (MS), although the causal relationship remains uncertain, as later highlighted in several meta-analyses (1–3). Important findings come from ‘The vitamin D and type 2 diabetes study (D2d)”, which was a randomized clinical trial (RCT) of overweight/obese participants in a prediabetes state, randomized to vitamin D_3–_4000 IU daily vs. placebo and followed for 2.5 years for the primary outcome of T2DM. This study revealed that an intra-trial mean serum 25 (OH) D level ≥ 40 ng/mL was associated with significantly reduced risk of T2DM, but only among participants with body mass index (BMI) < 40 kg/m^2^. The researchers suggested that targeting a serum 25 (OH) D level ≥ 40 ng/mL would be one of the T2DM prevention strategy in a prediabetic population with body mass excess (4).

Vitamin D deficiency exacerbates metabolic dysfunction through mechanisms involving insulin resistance (IR), inflammation, and dyslipidemia (5–7). Vitamin D is crucial for the regulation of insulin secretion in response to glucose and the modulation of hepatic glucose and triglyceride (TG) synthesis. It also contributes to reducing inflammation associated with IR, thereby lowering the risk of developing T2DM and cardiovascular disease (8–11). Moreover, vitamin D modulates intracellular calcium levels in pancreatic β-cells and hepatocytes, improving insulin sensitivity (12).

Deficiency of vitamin D exhibits pro-inflammatory activity and influences signaling pathways such as the receptor for advanced glycation end products (RAGE), promoting the development of both type 1 diabetes mellitus (T1DM) and T2DM (13). The discovery of 1,25(OH)_2_D_3_ and 1-α-hydroxylase expression in pancreatic β-cells further supports vitamin D’s role in glucose homeostasis (13). Vitamin D deficiency has also been linked to elevated inflammatory markers, including interleukin-6 (IL-6), tumor necrosis factor-α (TNF-α), and high-sensitivity C-reactive protein (hs-CRP), which contribute to T2DM pathogenesis. Vitamin D supplementation (VDS) has been shown to downregulate RAGE expression (13).

Previous studies have highlighted the difficulty of correcting vitamin D deficiency in obese individuals, who often exhibit reduced serum 25(OH)D concentrations as a result of adipose tissue sequestration, volumetric dilution, and decreased bioavailability (14–16). Pharmacokinetic evidence suggests that individuals with obesity require higher doses of vitamin D to achieve sufficient serum concentrations (17). Findings from RCTs and meta-analyses regarding changes in fasting serum glucose (FSG), glycated hemoglobin (HbA1c), body weight, insulin resistance (IR), and lipid profiles following VDS remain inconsistent (2, 3, 18–22). Chen et al. observed that young adults with vitamin D deficiency had a significantly higher risk of developing atherogenic dyslipidemia, indicating a potential role of vitamin D in the treatment of dyslipidemia (21). Similarly, it was highlighted that patients treated with statins who had low serum vitamin D levels had a higher risk of muscle pain (22).

Regarding oncological outcomes, meta-analyses of prospective cohort studies examining non-skeletal effects have demonstrated a significantly reduced risk of colorectal cancer among individuals with higher 25(OH)D concentrations, although this association has not been observed for most other malignancies (23). In an umbrella review, Liu et al. reported an association between vitamin D supplementation and lower all-cause mortality, but not with reduced risk of T2DM, Alzheimer’s disease, arterial hypertension, or schizophrenia (24).

Lifestyle interventions—particularly dietary counseling and structured physical activity—remain the cornerstone of T2DM and obesity management. Both approaches independently improve anthropometric, glycemic, and lipid outcomes (25, 26). One RCT in obese menopausal women reported that vitamin D_3_ supplementation enhanced the effects of supervised aquatic aerobic training on physical fitness indices compared with training alone, suggesting a potential synergistic effect on functional outcomes (27). However, whether VDS exerts additive or synergistic effects when combined with lifestyle measures remains uncertain.

The aim of this study was to evaluate the association between VDS and changes in anthropometric and selected metabolic parameters in ambulatory obese patients with T2DM.

## 2 Materials and methods

### 2.1 Study design and population

This 12-week prospective cohort study was conducted in Poland during the autumn–winter season (October 1, 2024 to March 31, 2025). Participants were recruited consecutively from a single outpatient metabolic clinic; all eligible patients attending routine follow-up visits during the recruitment period were screened and invited to participate, ensuring a non-selective recruitment process.

### 2.2 Attrition and follow-up

No participants were lost to follow-up. All 200 enrolled patients completed baseline and 12-week assessments and were included in the final analyses.

### 2.3 Seasonal control

Seasonal variability in endogenous vitamin D synthesis was minimized by conducting the entire study within a single autumn–winter period, when cutaneous vitamin D production is minimal at the study latitude.

### 2.4 Adherence assessment

Adherence to vitamin D supplementation was assessed using pill counts and self-reports. In addition, adherence was indirectly supported by the marked and consistent increase in serum 25(OH)D concentrations observed in the supplementation group.

### 2.5 Missing data

No missing data were observed for primary or secondary endpoints; therefore, no imputation procedures were required and all analyses were performed on complete cases.

### 2.6 Protocol and registration

The study protocol was predefined and approved by the institutional bioethics committee (see: 2.7 Ethical Approval). However, the study was not prospectively registered in a public registry, reflecting its observational design. This is acknowledged as a limitation.

### 2.7 Ethical approval

The decision to include a patient in the study was made by the attending physician. All recruited participants were fully informed about the study’s aims and conditions and provided written informed consent to participate. The study protocol was approved by the Bioethics Committee of the Poznan University of Medical Sciences (approval no. KB-664/23, September 13, 2023).

### 2.8 Inclusion criteria

Participants were eligible if they met the following criteria:

1. Age 18–64 years (young adulthood 18–39 years and middle adulthood 40–64 years)
2. Diagnosis of T2DM for at least 6 months according to the Polish Diabetes Association guidelines (28).
3. Baseline HbA1c between 6.5% and 7.5%.
4. Stable metformin monotherapy (1,500–2,000 mg/day) for at least 3 months before enrollment.
5. Serum calcium within the normal laboratory range.

### 2.9 Exclusion criteria

Patients were excluded for any of the following:

1. Current or recent (<6 months) use of vitamin D or calcium supplements.
2. Use of other antidiabetic agents (e.g., insulin, sulfonylureas, DPP-4 inhibitors, SGLT2 inhibitors, GLP-1 receptor agonists).
3. Advanced diabetic complications (e.g., nephropathy stage ≥3, proliferative retinopathy, or severe neuropathy).
4. History of hypercalcemia, nephrolithiasis, or parathyroid disorders.
5. Severe hepatic or renal impairment (eGFR <45 mL/min/1.73 m²).
6. Participation in another research study within the past 6 months.
7. Pregnancy or lactation.
8. Active malignancy or chemotherapy within 6 months.
9. Chronic liver disease (Child-Pugh class B or C) use of medications affecting vitamin D metabolism (anticonvulsants, glucocorticoids)

### 2.10 Intervention

Participants in the supplementation group received oral vitamin D_3_ - 4,000 IU daily for 12 weeks. The control group received no supplementation. Both cohorts continued stable metformin monotherapy throughout the study to ensure treatment uniformity. Adherence was verified by pill counts and self-reports.

### 2.11 Clinical endpoints

Primary endpoint:

• Change in serum 25(OH)D levels after 12 weeks.

Secondary endpoints:

- Changes in FSG, HbA1c, systolic and diastolic blood pressure (SBP, DBP), serum calcium, and BMI.
- Identification of predictors of failure to achieve HbA1c ≤6.5% (≤48 mmol/mol) after 12 weeks.

### 2.12 Measurements

Fasting blood samples were collected at baseline and after 12 weeks.

Laboratory Methods: All laboratory analyses were performed at the hospital’s certified clinical laboratory using standardized protocols.

Biochemical Measurements (29–31):

- 25-hydroxyvitamin D: Electrochemiluminescence immunoassay (Roche Cobas, Basel, Switzerland)
- Glucose: Hexokinase method (Roche Cobas)
- HbA1c: High-performance liquid chromatography (Bio-Rad Variant II, Hercules, CA)

Quality Control: Internal quality control samples were analyzed with each batch. External quality assurance was maintained through participation in national proficiency testing programs.

BP was measured in the seated position with an automated sphygmomanometer (average of three readings). BMI was calculated from measured weight and height.

### 2.13 Statistical analysis

Continuous variables are expressed as mean ± standard deviation (SD). Baseline group comparisons were performed with independent Student’s t-tests or chi-square tests for categorical variables. Within-group changes were analyzed using paired t-tests, and between-group differences in mean change (Δ) were compared with independent t-tests.

Multiple logistic regression identified predictors of failure to achieve HbA1c ≤6.5% at 12 weeks. Covariates included age (per 10 years), gender, FSG (per 2 mmol/L), BMI (per 5 kg/m²), and duration of T2DM (per 6 months). Results are presented as odds ratios (OR) with 95% confidence intervals (CI).

A two-sided p-value <0.05 was considered statistically significant. Analyses were performed using SPSS version 27.0 (IBM Corp., Armonk, NY, USA).

## 3 Results

### 3.1 Baseline characteristics

The study cohort included 200 patients with T2DM (51% men, 49% women) with a mean age of 58.6 ± 7.35 years and a mean BMI of 30.1 ± 3.5 kg/m². In the entire study cohort, the mean duration of T2DM was 1.4 ± 0.6 years, the mean HbA1c level was 6.8 ± 0.9%, and the mean FSG level was 5.8 ± 0.2 mmol/l. At baseline, mean serum 25(OH)D concentrations in both groups (~21–22 ng/mL) corresponded to vitamin D deficiency/insufficiency according to established clinical thresholds. The baseline clinical characteristics of the study participants and controls are presented in **Table 1**. No significant differences were observed between groups at baseline with respect to age, sex distribution, diabetes duration, serum 25(OH)D, FSG, HbA1c, BP, serum calcium, or BMI (all p > 0.5). (**Table 1**).

**Table 1:** Baseline clinical characteristics of study and control participants.

Parameter	Study group (n=100)	Control group (n=100)	p-value
Age (years)	58.4 ± 7.2	58.9 ± 7.5	0.68
Sex (male/female)	52/48	50/50	0.77
Duration of T2DM (years)	1.5 ± 0.6	1.3 ± 0.7	0.72
Serum 25(OH)D (ng/ml)	21.5 ± 6.8	22.1 ± 7.0	0.54
Fasting serum glucose (mmol/L)	5.8 ± 0.2	5.7 ± 0.2	0.81
HbA1c (%)	6.9 ± 1.0	6.7 ± 0.8	0.65
SBP (mmHg)	136.4 ± 12.1	135.9 ± 12.4	0.74
DBP (mmHg)	84.7 ± 7.9	84.9 ± 8.1	0.83
Serum calcium (mg/dL)	9.1 ± 0.4	9.2 ± 0.4	0.41
BMI (kg/m²)	30.2 ± 3.5	30.1 ± 3.6	0.82

### 3.2 Changes in selected metabolic parameters after 12 weeks of vitamin D3 supplementation in patients with T2DM

**Figure 1:**
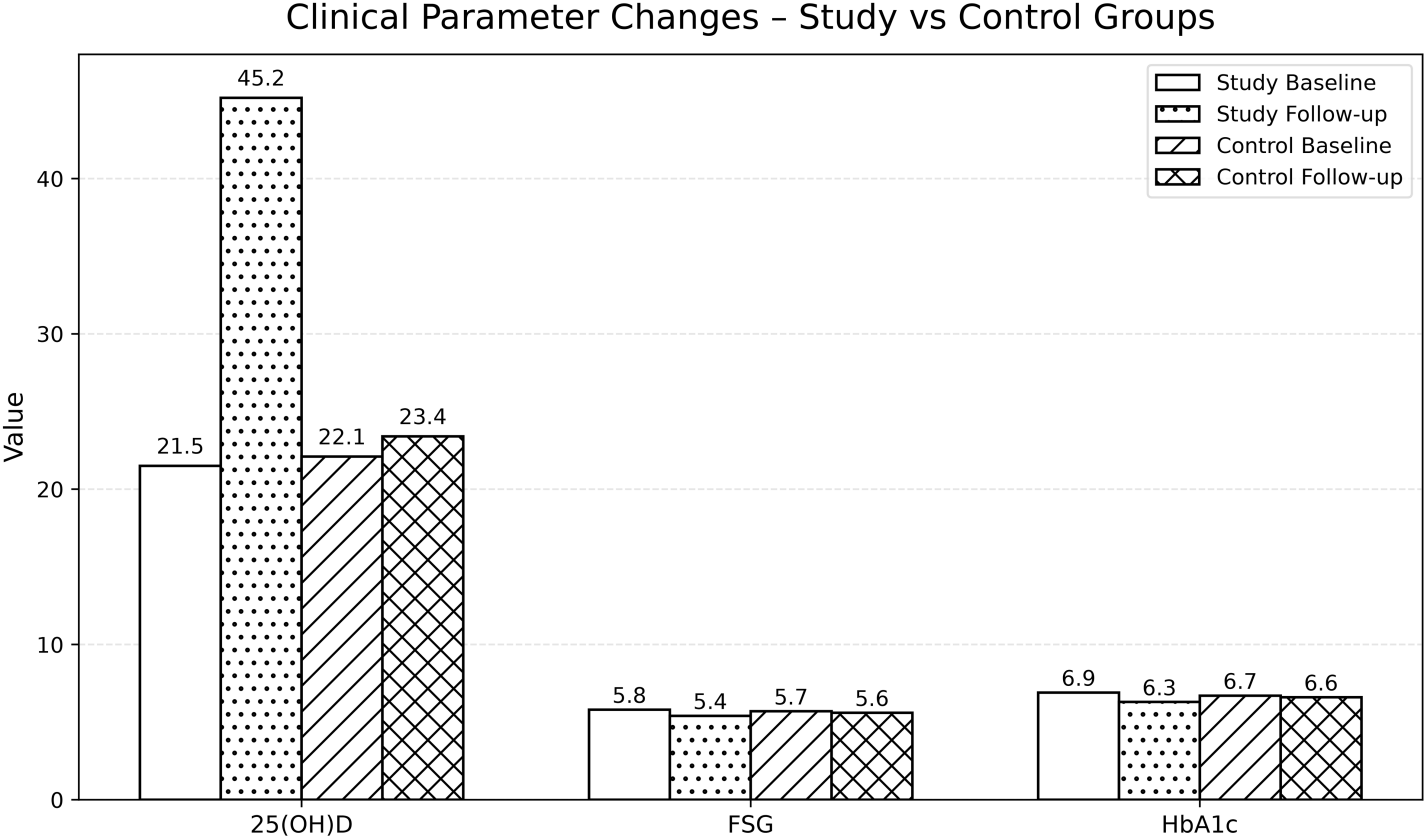
Baseline and follow-up clinical parameter levels in both groups.

**Figure 2:**
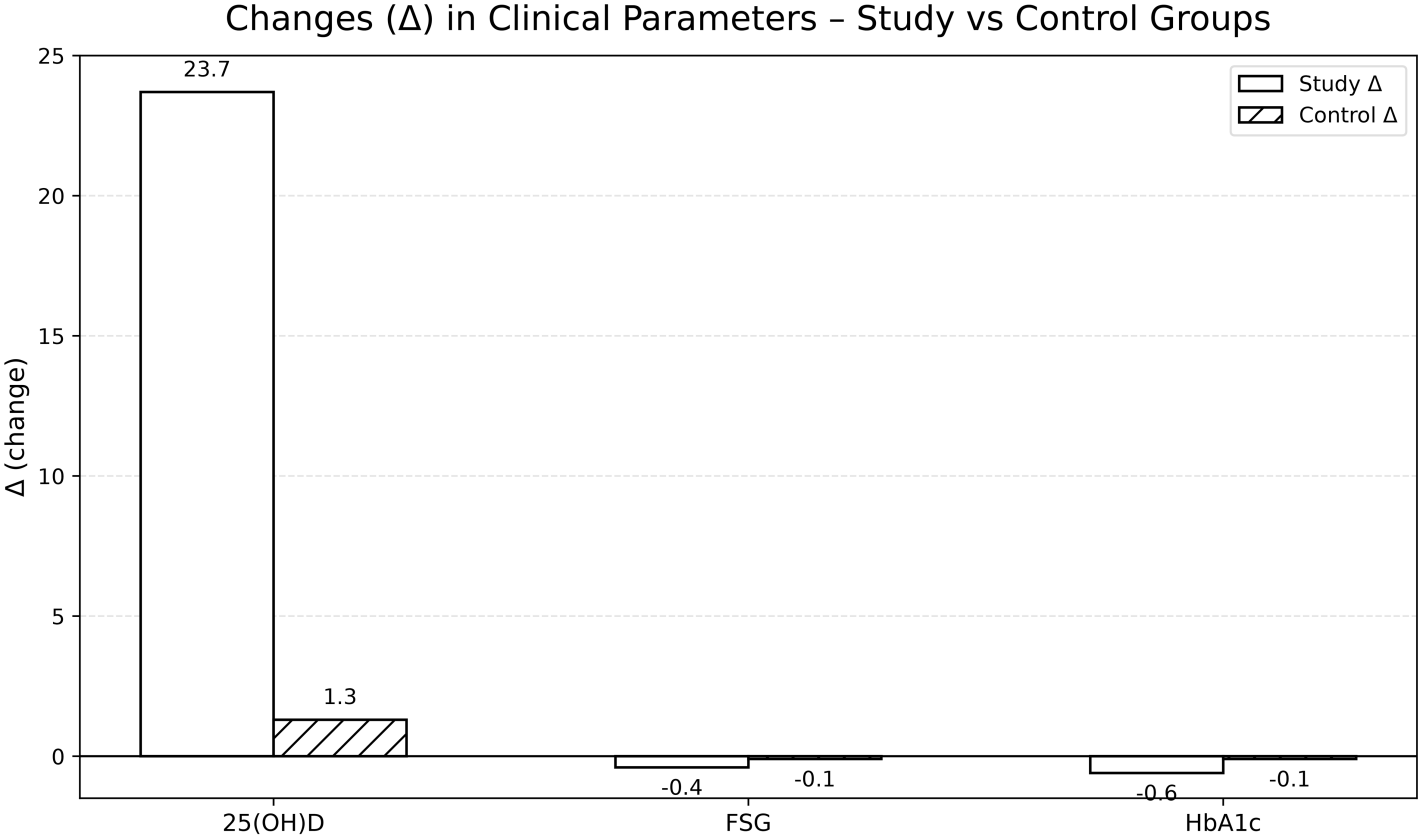
Comparative changes (Δ) in key clinical parameters: study vs. control group.

After 12 weeks, the supplementation group achieved a mean serum 25(OH)D concentration of approximately 45 ng/mL, corresponding to vitamin D sufficiency, whereas the control group remained within the deficient/insufficient range. Significant improvements were also observed in FSG and HbA1c, with mean reductions of –0.4 mmol/L and –0.6%, respectively, in the supplementation group. Changes in SBP and DBP, serum calcium, and BMI were not statistically significant (**Table 2**; **Figures 1**, **2**).

**Table 2:** Changes in selected metabolic parameters after 12 weeks of vitamin D3 supplementation in patients with T2DM.

Parameter	Study group – baseline	Study group – follow-up	Δ (change)	Control group – baseline	Control group – follow-up	Δ (change)	p-value
Serum 25(OH)D (ng/mL)	21.5 ± 6.8	45.2 ± 9.1	+23.7	22.1 ± 7.0	23.4 ± 7.5	+1.3	<0.001
FSG (mmol/L)	5.8 ± 0.2	5.4 ± 0.2	–0.4	5.7 ± 0.2	5.6 ± 0.2	–0.1	0.02
HbA1c (%)	6.9 ± 1.0	6.3 ± 0.8	–0.6	6.7 ± 0.8	6.6 ± 0.9	–0.1	0.01
SBP (mmHg)	136.4 ± 12.1	132.1 ± 11.5	–4.3	135.9 ± 12.4	135.5 ± 12.6	-0.4	0.06
DBP (mmHg)	84.7 ± 7.9	82.3 ± 7.5	–2.4	84.9 ± 8.1	84.4 ± 7.9	-0.5	0.07
Serum calcium (mg/dL)	9.1 ± 0.4	9.3 ± 0.5	+0.2	9.2 ± 0.4	9.2 ± 0.4	0.0	0.09
BMI (kg/m²)	30.2 ± 3.5	29.9 ± 3.4	–0.3	30.1 ± 3.6	30.2 ± 3.5	+0.1	0.12

Multiple logistic regression identified predictors of failure to achieve target HbA1c ≤ 6.5% (≤ 48 mmol/mol). Higher baseline FSG and longer T2DM duration were strongly associated with suboptimal glycemic control, with odds ratios (OR) of 1.34 (95% CI: 1.12–1.61; p = 0.001) and 1.28 (95% CI: 1.07–1.54; p = 0.006), respectively. Elevated BMI was also significantly associated with poor glycemic control (OR 1.21; 95% CI: 1.01–1.47; p = 0.04). In contrast, age and sex were not significant predictors (**Table 3**).

**Table 3:** Multiple logistic regression analysis of predictors associated with failure to achieve target HbA1c ≤ 6.5% (≤ 48 mmol/mol).

Predictor	Odds Ratio (OR)	95% CI	p-value
Age (per 10 years)	1.12	0.89 – 1.41	0.33
Gender (male vs female)	0.96	0.62 – 1.48	0.82
FSG (per 2 mmol/L)	1.34	1.12 – 1.61	0.001
BMI (per 5 kg/m²)	1.21	1.01 – 1.47	0.04
Duration of T2DM (per 6 months)	1.28	1.07 – 1.54	0.006

## 4 Discussion

In this 12-week prospective ambulatory cohort of patients with T2DM, high-dose VDS was associated with a marked rise in serum 25(OH)D concentrations and modest but statistically significant improvements in glycemic control (FSG and HbA1c), without significant effects on SBP, DBP, serum calcium, or BMI. Logistic regression confirmed that higher baseline FSG, longer T2DM duration, and higher BMI independently predicted failure to reach the HbA1c target ≤ 6.5%, whereas age and sex did not.

### 4.1 Interpretation of glycemic effects

The substantial increase in serum 25(OH)D among supplemented participants confirms effective absorption and bioavailability of the administered high-dose regimen. The observed reductions in FSG and HbA1c indicate that VDS was associated with modest improvements in glycemic parameters in patients with T2DM managed on stable metformin therapy.

These findings are consistent with several meta-analyses reporting modest but significant improvements in glycemic indices following VDS in diabetic or prediabetic populations. For example, Musazadeh et al. (2023) observed mean reductions in FSG (–3.08 mg/dL; 95% CI –3.97 to –2.19) and HbA1c (–0.05%; 95% CI –0.10 to –0.01) across 14 studies (17,136 participants) (32). Similarly, Farahmand et al. (2023) demonstrated significant decreases in FSG, HbA1c, and fasting insulin in RCTs, although the magnitude of effect was modest and heterogeneity was noted (2).

Conversely, other trials have failed to demonstrate significant HbA1c reductions, particularly in cohorts with sufficient baseline vitamin D levels or when using lower doses or shorter durations of therapy (33). Results of meta-analysis conducted by Qi et al. (2022) showed that VDS had no impact on HbA1c level and serum lipids, but reduced IR and BP values (34).

Although modest in magnitude, the observed HbA1c reduction (–0.6%) exceeds expected short-term biological variability and occurred in a population with relatively well-controlled T2DM at baseline, suggesting potential clinical relevance when interpreted as an adjunctive effect.

### 4.2 Interpretation of blood pressure results

In the present study, both SBP and DBP declined modestly in the intervention group but the changes were not statistically significant. Evidence regarding vitamin D’s influence on BP remains inconsistent. Lee and Lee (2016) reported reductions in DBP among patients with T2DM receiving vitamin D, whereas Jafari et al. (2018) found a significant reduction in SBP but not DBP (35, 36). As it was mentioned above, meta-analysis by Qi et al. (2022) showed the improvement in hypertension control in patients with VDS (34). In contrast, analyses in the general population have shown minimal or no effect of VDS on BP, suggesting limited causal influence (37). An umbrella meta-analysis by Meng et al. (2023) reported that the overall effects of vitamin D supplementation on blood pressure were modest (38).

Mechanistically, vitamin D may modulate BP through regulation of the renin–angiotensin–aldosterone system (RAAS), improvement of endothelial function, and enhanced vascular smooth muscle tone. These mechanisms may underlie variable BP responses among susceptible individuals (38).

### 4.3 Interpretation of body mass index results

Numerous systematic reviews, meta-analyses, and an umbrella review have examined the relationship between vitamin D and obesity, combining data from RCTs and cohort studies (4, 26, 39). The findings vary depending on factors such as population characteristics, initial vitamin D status, dosage, and accompanying interventions. Overall, meta-analytic and umbrella evidence suggests modest reductions in BMI and weight circumference (WC), though the effects on body weight and fat mass remain inconsistent. Multimodal strategies involving exercise, protein supplementation, or caloric restriction achieve more substantial and consistent improvements in body composition and function (40).

In the present study there was no significant change in BMI. One of the key factor is a short study period. In the meta-analysis conducted by Musazadeh et al. (2022), the effects of vitamin D on body weight and fat mass were not considerable, but VDS significantly improved BMI, and WC (39). Similarly, Hu et al. in an umbrella meta-analysis reported small pooled reductions in BMI and WC, but no meaningful weight or fat−mass change overall (26).

In turn, Aquino et al. (2023) did not show a decrease of WC in patients with MS (41). Roth et al. (2022) reported that the effect of VDS on the preservation of free fat mass (FFM) during non-surgical and surgical weight loss remains controversial (40).

### 4.4 Predictors of glycemic non-response

Logistic regression analysis identified higher baseline FSG, longer disease duration, and higher BMI as significant predictors of failure to achieve target HbA1c levels. These findings are physiologically plausible and consistent with clinical experience, as patients with more pronounced baseline hyperglycemia, longer disease duration, or obesity typically exhibit reduced responsiveness to metabolic interventions. The lack of association with age and sex suggests that metabolic factors, rather than demographic characteristics, primarily determine glycemic response.

### 4.5 Strengths and limitations

This study offers several methodological strengths. The use of a standardized high-dose vitamin D regimen achieved robust improvement in serum 25(OH)D concentrations, enabling clear evaluation of metabolic effects. Homogeneous treatment with metformin monotherapy minimized pharmacologic confounding. The prospective, ambulatory design enhances external validity and clinical relevance, while multivariable modeling accounted for key covariates, strengthening internal validity. Seasonal variation in 25(OH)D concentrations could not have influenced the results, as the study was conducted during the autumn–winter 2024/2025 season.

Nonetheless, limitations must be acknowledged. The non-randomized design introduces potential residual confounding, including behavioral and unmeasured factors. The 12-week duration limits assessment of long-term outcomes. The sample size, although moderate, may have been underpowered to detect small changes in calcium or BMI. Finally, findings may not generalize to patients using complex or combination antidiabetic regimens.

The observational design precludes causal inference. Potential residual confounding, lack of placebo control, reliance on pragmatic adherence measures, and absence of prospective registration are acknowledged limitations. Observational designs often underestimate the true effect of vitamin D due to seasonal and temporal variability in serum levels. As emphasized by Pilz et al. (2022), large RCTs have generally failed to demonstrate consistent reductions in adverse outcomes, possibly owing to study design and dosing heterogeneity (42). Observational studies allowing variable supplementation and repeated 25(OH)D measurements may provide broader insight into dose–response relationships and health outcomes (43, 44).

Proposed biological mechanisms discussed herein are derived from prior experimental and clinical studies and were not directly investigated in the present study. They are included to provide biological plausibility and should be considered hypothesis-generating.

### 4.6 Implications and future directions

The present findings support the hypothesis that high-dose VDS may offer adjunctive benefits in glycemic control among obese T2DM patients with baseline deficiency. However, due to the observational nature of this study, causality cannot be inferred. Future RCTs with longer follow-up, uniform treatment regimens, and stratification by baseline vitamin D status and body composition are warranted. Mechanistic studies exploring effects on insulin sensitivity, β-cell function, inflammation, RAAS activity, and endothelial function would further elucidate the metabolic and cardiovascular implications of vitamin D in T2DM and obesity.

Recent research suggests that vitamin D supplementation may offer beneficial effects on oxidative stress as well as on reproductive, endocrine, and metabolic functions (45). In Hashimoto’s thyroiditis VDS shows promise in influencing immune activity, reducing symptom severity, and enhancing overall quality of life (46). Further investigation is needed to clarify the full range of potential therapeutic applications of VDS in diabetic neuropathy and statin-associated muscle symptoms (SAMS) (47, 48). So far, evidence suggest that vitamin D may support fertility and overall metabolic well-being by modulating hormone concentrations and key metabolic indicators (45). Despite these encouraging findings, additional studies are required to clarify the most effective dosage, treatment length, and timing for supplementation. It is essential for scientists, healthcare professionals, and policy leaders to work together to develop clear, evidence-based recommendations for the use of vitamin D in above mentioned indications (45–48).

### 4.7 Conclusions

High-dose VDS was associated with improved vitamin D status and modest improvements in glycemic control in obese patients with T2DM. These findings should be interpreted as associative rather than causal and support further investigation in randomized, placebo-controlled trials with extended follow-up and mechanistic endpoints.

## Data Availability

The raw data supporting the conclusions of this article will be made available by the authors, without undue reservation.
